# Monocyte Exosomes Stimulate the Osteogenic Gene Expression of Mesenchymal Stem Cells

**DOI:** 10.1371/journal.pone.0075227

**Published:** 2013-09-18

**Authors:** Karin Ekström, Omar Omar, Cecilia Granéli, Xiaoqin Wang, Forugh Vazirisani, Peter Thomsen

**Affiliations:** 1 Department of Biomaterials, Institute of Clinical Sciences, Sahlgrenska Academy at University of Gothenburg, Gothenburg, Sweden; 2 BIOMATCELL VINN Excellence Center of Biomaterials and Cell Therapy, Gothenburg, Sweden; Rutgers - New Jersey Medical School, United States of America

## Abstract

Inflammation and regeneration at the implant-bone interface are intimately coupled via cell-cell communication. In contrast to the prevailing view that monocytes/macrophages orchestrate mesenchymal stem cells (MSCs) and progenitor cells via the secretion of soluble factors, we examined whether communication between these different cell types also occurs via exosomes. LPS-stimulated human monocytes released exosomes, positive for CD9, CD63, CD81, Tsg101 and Hsp70, as determined by flow cytometry and Western blot. These exosomes also contained wide size distribution of RNA, including RNA in the size of microRNAs. The exosomes were shown to interact with human mesenchymal stem cells. After 24 h of culture, a considerable portion of the MSCs had internalised PKH67-labelled exosomes. Furthermore, after 72 h, the gene expression of the osteogenic markers runt-related transcription factor 2 (RUNX2) and bone morphogenetic protein-2 (BMP-2) had increased in comparison with control medium, whereas no significant difference in osteocalcin (OC) expression was demonstrated. The present results show that, under given experimental conditions, monocytes communicate with MSCs via exosomes, resulting in the uptake of exosomes in MSCs and the stimulation of osteogenic differentiation. The present observations suggest that exosomes constitute an additional mode of cell-cell signalling with an effect on MSC differentiation during the transition from injury and inflammation to bone regeneration.

## Introduction

Exosomes are nano-sized extracellular vesicles (EV) involved in the communication between cells. Exosomes are formed within endosomal compartments and released into the extracellular environment [1]. Exosomes are released from many cells, including dendritic cells [2], mast cells [3], epithelial cells [4], tumour cells [5], macrophages [6] and stem cells [7], [8]. Exosomes are also present in biological fluids, such as blood plasma [9], amniotic fluid [10], saliva [11], nasal lavage fluid [12], urine [13], breast milk [14] and cerebrospinal fluid [15]. Exosomes are regarded as powerful mediators of cell-cell communication due to their ability to shuttle functional RNA and proteins between cells [16], [17]. In relation to regenerative processes, exosomes from CD34^+^ hematopoietic progenitors have been shown to induce angiogenesis both *in vitro* and *in vivo* [8]. In addition, microvesicles and exosomes from an lipopolysaccharide (LPS)-activated monocyte cell line (THP-1) have been shown to regulate the gene expression in recipient endothelial cells, increasing their thrombogenicity and apoptosis [18].

Following the implantation of titanium implants in bone, both monocytes and regenerative mesenchymal stem cells (MSCs) are recruited and accumulated at the interface [19]. It is suggested that the early monocyte activity at the interface influences the neighbouring MSCs, affecting the MSC differentiation and regenerative capacity. This interaction subsequently influences the amount of bone formation and the implant stability in the recipient bone site [20]. In an *in vitro* model, we have recently found that conditioned medium (CM) from classically activated monocytes transferred to undifferentiated MSCs up-regulates the expression of genes involved in osteogenic differentiation in the recipient cells [21]. Immunoassays of the CM revealed high levels of pro-inflammatory cytokines, tumour necrosis factor-alpha (TNF-α) and monocyte chemotactic protein-1 (MCP-1), but failed to detect the strongly pro-osteogenic factor bone morphogenetic protein 2 (BMP-2). It is therefore possible that the osteogenic signal transferred from monocytes to MSCs, via the CM, involves other mechanisms, possibly in parallel with the secretion of soluble mediators in the CM.

By virtue of their versatility and their early co-existence with MSCs at the implant-bone interface, we hypothesised that monocytes release exosomes with regenerative potential that can influence the MSC osteogenic differentiation. To address the hypothesis, the study aimed firstly to examine whether classically activated primary human monocytes release exosomes and whether human MSCs internalise these exosomes. Secondly, the study aimed to evaluate the osteogenic gene expression in the MSCs after exposure to exosomes from the classically activated monocytes.

## Materials and Methods

### Isolation and culture of human primary cells

The mesenchymal stem cells were isolated from bone marrow from iliac crest obtained from donors undergoing surgical spinal fusion at the Sahlgrenska University Hospital (Gothenburg, Sweden). The isolation, characterisation and culture of the MSCs were performed as described elsewhere [21].

Monocytes were isolated from human blood using a two-step procedure. Firstly, peripheral blood mononuclear cells (PBMCs) were obtained by Ficoll-Paque density separation using Leucosep® tubes (Greiner BioOne GmbH, Frickenhausen, Germany), according to the manufacturer’s protocol. The PBMCs were washed repeatedly with 2 mM EDTA in PBS and then dissolved in 0.5% BSA/2 mM EDTA in PBS. Secondly, the monocytes were isolated from the PBMCs by negative isolation using a Monocyte Isolation kit II (Miltenyi Biotec, Bergisch Gladbach, Germany), according to the manufacturer’s instructions. The purity of the separations ranged between 90% and 95%, as analysed by flow cytometry using antibodies against CD14 and CD45 or isotype-matched controls (BD Biosciences, San Diego, CA, USA) in a BD FACSCalibur (Becton Dickinson, San Diego, CA, USA). The monocytes were cultured for 72 h in a 37°C humidified incubator with 5% CO_2_, at a concentration of 5×10^5^ cells/ml in Dulbecco’s modified Eagles medium-low glucose (DMEM-LG) (PAA Laboratories GmbH, Pasching, Austria) containing 10 ng/ml lipopolysaccharide (LPS; Escherichia coli serotype, Sigma Aldrich, St Louis, MO, USA, batch number 126k4101), 1% fetal calf serum (FCS), 100 units/ml penicillin, 100 µg/ml streptomycin and 2 mM L glutamine (all from Sigma-Aldrich). In order to have exosome-free serum for the cultures, FCS was centrifuged at 150,000 g for 2 h using a Ti70 rotor (Beckman Coulter, Brea, CA, USA). Serum depleted of exosomes was used for all cell culture experiments.

### Collection of monocyte conditioned medium and isolation of exosomes

After 72 h, CM was collected, centrifuged at 500 g for 10 min to eliminate cells and saved at –70°C until exosome isolation. Part of the CM was saved for the subsequent culture of MSCs. The other part was used to isolate and characterise exosomes. To isolate exosomes, CM depleted of cells was centrifuged at 16,500 g for 20 min, followed by filtration through a 0.22 µm filter to remove cell debris. Exosomes were pelleted by ultracentrifugation (Beckman Ti70 rotor) at 120,000 g for 70 min. For PKH67 transfer experiments, exosomes were dissolved in PBS and centrifuged a second time at 120,000 g for 70 min. All steps were performed at 4°C.

### Flow cytometry of monocyte exosomes

For the coating of latex beads, 4 µm-diameter aldehyde/sulphate latex beads (Interfacial Dynamics, Portland, OR, USA) were incubated with purified anti-CD63 antibody (BD Biosciences) under agitation at RT overnight, according to the manufacturer’s protocol. For FACS analysis, 5 µg of monocyte exosomes were incubated with 1.5×10^5^ anti-CD63 beads in 50 µl PBS at RT for 15 min. The volume was made up to 300 µl and the beads were incubated at 4°C overnight under gentle agitation. The reaction was stopped by incubation in 100 mM glycine for 30 min. Exosome-coated beads were washed twice, incubated in 50 µg human IgG (Sigma-Aldrich) at 4°C for 15 min, washed twice and incubated with anti-CD9 FITC, anti-CD63 PE, anti-CD81 APC or matching isotype controls (BD Biosciences), washed twice and acquired using a FACSCalibur (BD Biosciences). Ten thousand events were acquired using CellQuest and the data were analysed using FlowJo Software (Tree Star, Inc, Ashland, OR, USA).

### Western blot analysis of monocyte exosomes

Cellular and exosomal proteins were extracted using M-per®Mammalian Protein Extraction Reagent containing protease inhibitors (Thermo Scientific, USA). The samples were incubated for 10 min on a shaker before centrifugation at 14,000 g for 10 min. The protein concentration of the supernatant was determined using a Pierce®BCA protein Assay kit (Thermo Scientific). Equal amounts of proteins (5 µg) were loaded per well and separated on a 10% Mini-Protean®TGX Precast Gel (Bio-Rad Laboratories) and transferred to PVDF membranes (Bio-Rad Laboratories). To prevent non-specific binding, membranes were blocked with 0.5% non-fat milk powder in Tris-buffered saline (TBS)-Tween at RT for 2 h. The membranes were then incubated with rat monoclonal anti-Hsp70 [clone 1B5] (heat shock protein 70; diluted 1∶1000, Enzo Life Sciences, Exeter, UK), mouse monoclonal anti-Tsg101 [clone 4A10] (Tumour susceptibility gene 101 protein; diluted 1∶500, Abcam, Cambridge, UK), rabbit polyclonal anti-calnexin [clone H-70] (diluted 1∶1000, Santa Cruz Biotechnology, Santa Cruz, CA, USA) and rat monoclonal anti-Grp94 [clone 9G10] (Glucose-regulated protein 94; diluted 1∶1000, Enzo Life Sciences) at RT for 2 h with gentle shaking. All antibody dilutions were made in 0.25% non-fat milk powder in TBS-Tween. After incubation with primary antibodies, membranes were washed for 3×10 min in TBS-Tween (used for all wash steps) before incubation with secondary horseradish peroxidase (HRP)-conjugated antibodies for 2 h at RT. The secondary antibodies used were goat anti-rat IgG (diluted 1∶10,000, Enzo Life Sciences), goat anti-mouse IgG (diluted 1∶2000, Santa Cruz) and goat anti-rabbit IgG (diluted 1∶10,000, Santa Cruz). The membranes were washed 3×15 min, and finally developed using the Immun-Star™WesternC™ chemiluminescence detection kit (Bio-Rad Laboratories), according to the instructions of the manufacturer. Digital detection was made using the ChemiDoc XRS+ system with Image Lab Software (Bio-Rad Laboratories).

### Exosomal RNA isolation and detection

RNA from isolated exosomes and LPS-stimulated monocytes was extracted using a miRCURY™ RNA Isolation Kit (Exiqon, Vedbaek, Denmark), according to the manufacturer’s protocol. Samples were DNase treated for 15 min using an RNase-free DNase kit (QIAGEN GmbH, Hilden, Germany), according to the manufacturer’s protocol. The detection and quality control of RNA was performed using an Agilent 2100 Bioanalyzer (Agilent Technologies, Foster City, CA, USA) for nano, pico and small RNA profiles, according to the manufacturer’s protocol.

### Mesenchymal stem cell uptake of the monocyte exosomes

For uptake experiments, MSCs at passage 5–7 (n = 3) were seeded in 24-well plates or chamber slides (LAB-TEK Nunc) at a density of 10,000 cells/cm^2^ and cultured for 24 h. Monocyte exosomes were labelled with a PKH67 Green Fluorescent Cell Linker Kit for General Cell Membrane Labelling (Sigma-Aldrich), according to the manufacturer’s protocol with some modifications. Briefly, 1350 µl of Diluent C was added to 150 µl of exosomes or PBS control (no exosomes). Six µl of PKH67 dye was added to 1.5 ml of Diluent C before being added to the exosomes and the control. The samples were mixed gently for 5 min before 3 ml of 1% BSA was added to bind the excess dye. The samples were then transferred to 300 kDa Vivaspin filters (Sartorius Stedim Biotech GmbH, Goettingen, Germany) and centrifuged at 4000 g. The samples were washed with 5 ml 1% BSA/PBS, transferred to new 300 kDa filters and washed twice with 5 ml 1% BSA/PBS and once with 5 ml DMEM to remove excess dye. After washing, the exosomes and PBS control were diluted in complete monocyte culture medium (DMEM containing 1% depleted FCS, 1% penicillin-streptomycin, 2 mM L-glut and 10 ng/ml LPS). The medium was aspirated and PKH67 stained exosomes or control was added. After 72 h, cells were harvested and the binding of the exosomes to the MSCs was analysed with a FACSCalibur or visualised using fluorescence microscopy (Nikon Eclipse E600, Tokyo, Japan) connected to a personal computer with ACT-1 (Ver2.70) software. For analysis with flow cytometry, cells were washed twice with PBS, scraped, washed twice with 1% FCS/PBS and fixed in 1% formaldehyde before being acquired in a FACSCalibur and analysed with FlowJo Software (Tree Star, Inc). For fluorescence microscopy, the cells were washed twice with PBS, fixed with 2% formaldehyde for 15 min and washed twice in PBS before being mounted with VECTASHIELD HardSet Mounting Medium with DAPI (Vector Laboratories, Burlingame, USA).

### Gene expression analysis of mesenchymal stem cells cultured with monocyte exosomes

For gene expression analysis experiments, MSCs were seeded at a density of 8000 cells per cm^2^ and cultured in normal expansion medium. When the cells reached ∼80% confluence, the expansion medium was aspirated and changed to monocyte CM, exosome-enriched medium or control medium. After 72 h of culture, the RNA was extracted using an RNeasy Plus Micro Kit (QIAGEN), according to the manufacturer’s protocol. DNase treatment was performed in order to eliminate any contaminating genomic DNA. The quantification of nucleic acids was performed using a NanoDrop ND-1000 spectrophotometer (Thermo Scientific) and the quality of the RNA was verified using an Agilent Bioanalyzer with an RNA 6000 Nano Assay (Agilent Technologies). RNA was transcribed to cDNA using a High Capacity RNA-to-cDNA Kit (Applied Biosystems, California, USA). Real-time PCR was carried out using cDNA equivalent to 2.5 ng RNA and the TaqMan® Universal PCR Master Mix with Assays-on-demand™ mixes of primers and TaqMan MGB probes (Applied Biosystems). All samples were analysed in duplicate and PCR was performed using the 7900HT real time PCR System (Applied Biosystems). The assays used in this study were RUNX2 (Hs00231692_m1), BMP-2 (Hs00154192_m1), OC (Hs01587813_g1) and 18S (Hs99999901_s1) as reference gene (Applied Biosystems). No reverse transcriptase and no template controls were included as negative controls. The relative quantification of the target gene expression was calculated by the delta delta-Ct method.

### Statistics

The Kruskal Wallis test followed by the Mann-Whitney test was used for the statistical analysis and a p value of <0.05 was considered significant.

### Ethics Statement

The mesenchymal stem cells were isolated from bone marrow from the iliac crest obtained from donors undergoing surgical spinal fusion at the Sahlgrenska University Hospital (Gothenburg, Sweden) under ethical approval (532-04) from the Regional Ethical Review Board, University of Gothenburg. Written consent was collected for bone marrow donors. The consent form is part of the ethics application that was approved by the committee. Human blood was obtained from the blood bank at Sahlgrenska University Hospital, Gothenburg, Sweden.

## Results

### Characterisation of monocyte exosomes

**Figure 1:**
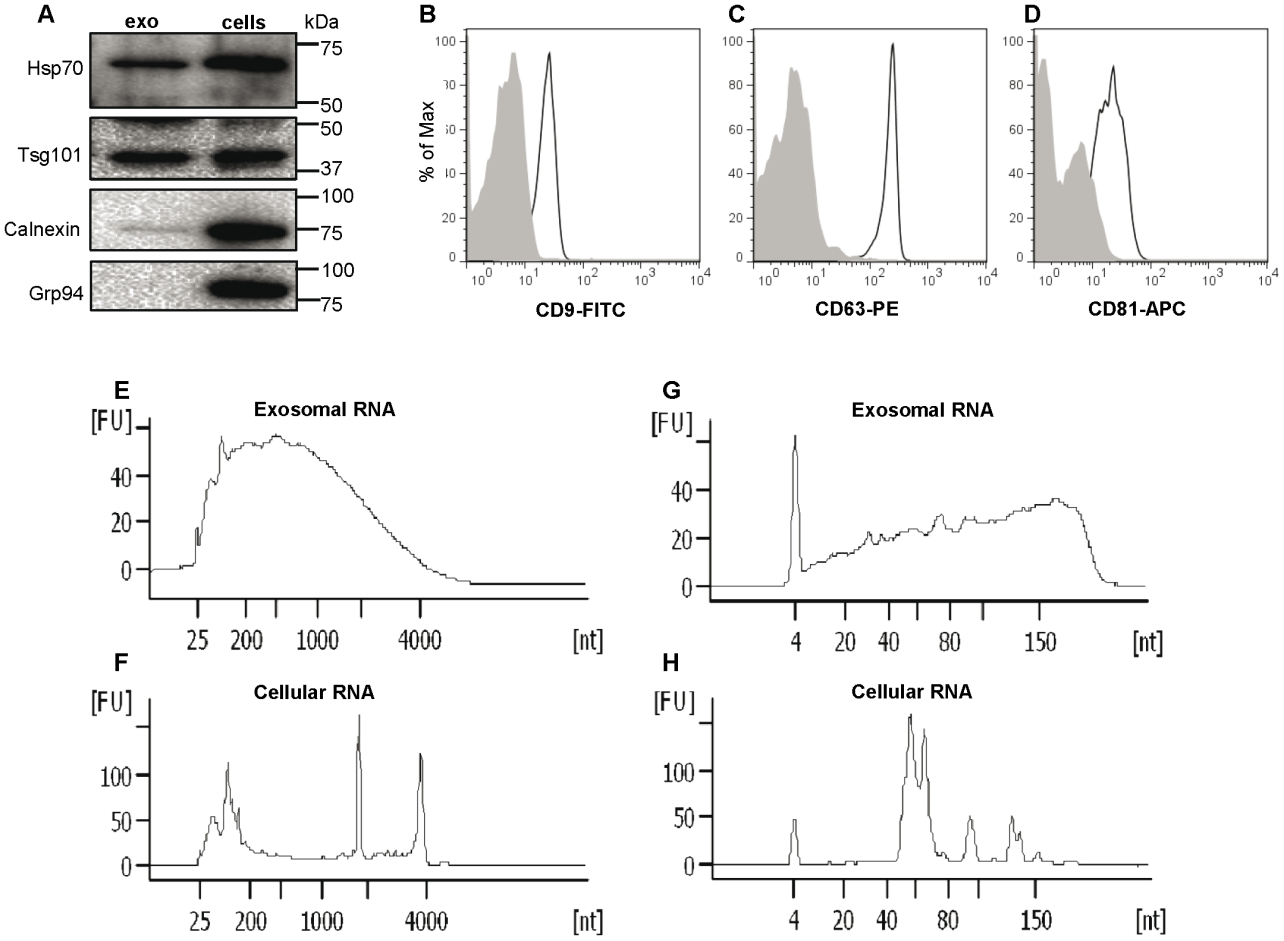
Human LPS-stimulated monocytes release exosomes that contain RNA. (a) Western blot for the exosome-associated proteins Hsp70 and Tsg101 and the endoplasmic reticulum proteins calnexin and Grp94 in LPS-stimulated monocytes (cells) and exosomes isolated from the monocytes (exo). The exosomes are positive for the exosome-associated proteins Tsg101 and Hsp70, while little or no endoplasmic reticulum protein calnexin and Grp94 is detected. (b-d) Flow cytometry detection of monocyte-derived exosomes captured on anti-CD63 coated latex beads. The exosome-bead complexes were immunostained against the tetraspanins CD9 (b), CD63 (c) and CD81 (d) (open curves) or their corresponding isotype controls (filled curves). (e-h) Bioanalyzer analysis of cellular and exosomal total and small RNA. The electropherograms show the size distribution in nucleotides (nt) and fluorescence intensity (FU) of total RNA in (e) exosomes and cells (f) and small RNA in exosomes (g) and cells (h).

Exosomes were isolated from the CM of LPS-stimulated human primary monocytes through a series of centrifugation and filtration steps. The presence of exosomes was determined by Western blot and flow cytometry. Western blotting for the exosome-associated proteins Tsg101 and Hsp70 and endoplasmic reticulum proteins Grp94 and calnexin revealed that the exosomes were positive for Tsg101 and Hsp70, while no or small amounts of Grp94 and calnexin were detected (Fig. 1a). Flow cytometric analysis of exosomes captured onto anti-CD63 latex beads revealed that they were positive for the tetraspanins CD9, CD63 and CD81 (Fig. 1b-d). Together, this verifies that the studied vesicles are exosomes. The RNA profile of exosomes and cells was examined using capillary electrophoresis in a Bioanalyzer (Fig. 1e-h). Bioanalyzer electropherograms of exosomal (Fig. 1e) and cellular (Fig. 1f) total RNA show the enrichment of ribosomal RNA in cells, which is not seen in the exosomes. Exosomes released from LPS-stimulated human monocytes were shown to contain RNA with a wide size distribution, which has previously been demonstrated in exosomes from other sources. The electropherograms for the small RNA show that both exosomes and cells contain small RNA, including RNA in the size of microRNA (Fig. 1g-h).

### Exosome uptake by mesenchymal stem cells

**Figure 2:**
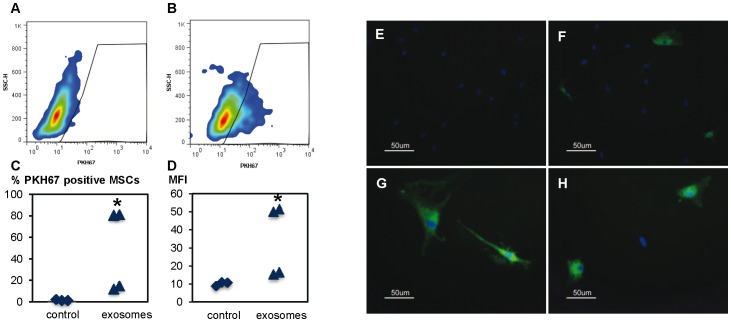
Uptake of monocyte-derived exosomes by MSCs. PKH67-labelled (green) monocyte exosomes or PKH67 control were added to MSCs in culture and the uptake was examined after 72 h using flow cytometry (a-d) and fluorescence microscopy (e-h). (a) Shows MSCs cultured with PKH67 control, (b) MSCs cultured with PKH67-labelled exosomes, (c) the percentage of MSCs positive for the PKH67 stain after 72 h and (d) the mean fluorescence intensity of the MSCs after co-culture with the green exosomes or control (n = 4, *p<0.05). (e-h) Fluorescence micrograph of MSCs (blue nucleus stained with DAPI). (e) MSCs co-cultured with control PKH67. (f-h) MSCs co-cultured with exosomes (green) show that some cells are positive for the exosomes while others are negative. (g) The intensity of the staining varies between cells.

To examine whether monocyte-derived exosomes interact with MSCs, fluorescent-labelled exosomes were incubated with MSCs, for 72 h, and examined using flow cytometry and fluorescence microscopy. Using flow cytometry, exosomes were shown to interact with the MSCs, where 46%±20% of the MSCs were positive for the PKH67 (Fig 2a-d, n = 4). The quantitative flow cytometry data, on the exosome-MSC interaction, were further supported by the fluorescence microscopy observations showing co-localisation of the PKH67-stained exosomes and the MSCs (Fig. 2e-h). Under the fluorescence microscope, while some MSCs were positive for the stained exosomes, other cells, in the same culture, did not show any fluorescence, indicating that only a fraction of the MSCs are positive for the green-labelled exosomes. Furthermore, the intensity of the PKH67 staining of the positive MSCs appeared to vary between cells in the same culture (Fig. 2b and g).

### Gene expression analysis of MSCs cultured with monocyte exosomes

**Figure 3:**
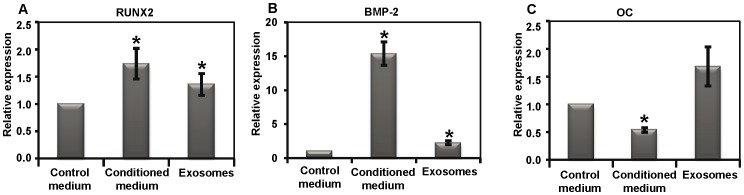
Osteogenic gene expression in MSCs. Gene expression for RUNX2 (a), BMP-2 (b) and OC (c) in MSCs, cultured in unconditioned control medium, in monocyte-conditioned medium (CM) or in medium supplemented with exosomes isolated from the CM for 72 h (n = 4). Each bar represents the mean ± SEM, n = 4. *p<0.05 compared with the medium control.

Since MSCs were shown to interact with monocyte exosomes, we aimed to determine whether exosomes are able to alter the osteogenic gene expression in the MSCs. To examine this, MSCs were cultured, for 72 h, under three different conditions: i) with exosomes isolated from the monocyte CM, ii) with full monocyte CM and iii) with control regular culture medium, after which osteogenic gene expression was analysed. The culture of MSCs in monocyte CM resulted in significantly fold higher expression of RUNX2 (1.7±0.3) and BMP-2 (15.4±1.7), as compared to the control media (Fig. 3a-b). This increased expression of RUNX2 and BMP-2 was also revealed when adding isolated exosomes to the MSCs (RUNX2 1.4±0.2 and BMP-2 2.3±0.3, respectively). The expression of OC showed a trend for higher expression in the MSCs receiving exosomes (1.7±0.4), while the expression was significantly decreased when the MSCs were cultured in the full CM (0.5±0.0, Fig 3c).

## Discussion

In the present study, a novel mode of communication between primary human monocytes and MSCs is described, with the emphasis on pro-osteogenic effects. We show that exosomes, released from LPS-activated monocytes, affect MSCs, resulting in increased osteogenic gene expression in the MSCs. It was recently reported that CM from classically (LPS stimulation), but not alternatively (IL-4 stimulation), activated monocytes stimulated osteogenic gene expression in MSCs [21]. In the present study, it was revealed that a substantial part of the osteogenic effects of the CM is mediated by exosomes.

Monocyte- and macrophage-derived exosomes have previously been described, with the emphasis on bacteria, viruses and prions, in addition to their potential use as vectors for gene delivery [6], [18], [22]–[24]. In most of these studies, cell lines are used. In the present study, we chose to investigate exosomes from primary human LPS-activated monocytes as we have previously reported an effect of monocyte CM on the osteogenic differentiation of MSCs. The aim in the present work is to answer the question of whether exosomes could play a mechanistic role in such effect. The decision to employ LPS as the trigger molecule for monocytes does not exclude the fact that there is also an urgent need systematically to investigate the release, content and function of exosomes from monocytes exposed to other types of stimulus, e.g. alternative IL-4 stimulation, or cultured on biomaterials with different surface properties.

For exosome detection and characterisation, a combination of methods is often recommended, as there are as yet no known exosome-specific markers. In general, exosomes can be detected based on their size, density and absence/presence of specific proteins. The level of cellular contamination can be estimated using antibodies against proteins that are not supposed to be sorted into exosomes, such as endoplasmic reticulum (ER), nucleus or mitochondria proteins. In this study, the presence of two ER proteins (calnexin and Grp94) was examined. A low level of calnexin was found, whereas Grp94 was absent in the exosome samples. The presence of CD9, CD63, CD81, Tsg101 and Hsp70, in combination with the absence of Grp94, strongly suggests that the studied vesicles are exosomes. It is interesting to note that, when examining the exosomes from LPS-activated monocytes isolated by repeated centrifugation and filtration steps followed by ultracentrifugation, one of the markers (calnexin) is slightly positive, while the other (Grp94) is negative. The low level of calnexin in the exosome samples might indicate a slight degree of contamination with other vesicles such as ER vesicles. Alternative isolation methods, such as density gradients by sucrose gradients or cushions, immune separation using magnetic beads or chromatography, need to be used when it is important to obtain exosomes with high purity. These methods are therefore commonly used when the downstream analysis requires a pure exosome sample. However, for functional assays, it is equally important that the isolated exosomes are intact and functional and filtration and ultracentrifugation methods are therefore useful. In fact, Welton *et al*. detected calnexin in exosomes isolated from cancer cells. However, using sucrose gradients, it was shown that most of the calnexin was not localised to the same fractions as the exosome marker proteins [25].

In recent years, the role of exosomes in cell-to-cell communication has attracted a great deal of interest and many cell types have been shown to communicate via exosomes. Since it was shown previously that monocytes and MSCs communicate in the absence of direct cell-to-cell contact [21] and that monocytes release exosomes, we examined whether monocyte-derived exosomes are able to interact with MSCs. Using PKH67 labelling, flow cytometry and fluorescence microscopy, the present results show that MSCs internalise monocyte-derived exosomes. Interestingly, the uptake of exosomes appears to vary between cells within the same culture and also between different experiments. The reason for this observation has not been further examined in this study, but, speculatively, the possibility cannot be excluded that the recipient MSCs differ either in their proliferation status or in the content of membrane receptors. In addition, there might be a range of specific structural differences between the exosomes, such as surface receptors or ligands, which may induce the observed differential interaction with the MSCs. Further investigations will therefore be important in order to address the question of whether differences in uptake by MSCs are related to different phenotypic characteristics of the MSCs. Moreover, albeit speculative, the possibility cannot be excluded that exosomes could have been internalised and subsequently have been degraded (before the 72 h observation time) or even “re-shuttled”.

Since the exosomes isolated from the monocyte CM were shown to interact with the MSCs, we further examined whether the monocyte-derived exosomes would induce osteogenic gene expression in the MSCs. To confirm the cell-system model, a full monocyte CM was included as a positive control. As in our previous study [21], the culture of MSCs in CM from LPS-stimulated monocytes resulted in the increased expression of RUNX2 and BMP-2, confirming the reproducibility of the model. Interestingly, for the osteogenic key regulator, RUNX2, the gene expression level was comparable to that induced by the CM, suggesting that the exosomes possess the same potential to steer the MSCs toward the osteogenic pathway as that provided by a full CM. On the other hand, the expression of BMP-2 was lower after exposure to exosomes compared with the full CM, but still higher than the control, unconditioned medium. The lower effect of exosomes, compared with the full CM, might be attributed to the loss of some exosomes and/or their functional effect, during the isolation procedure. It is worth noting that, in the present study, equal volumes of the CM were used to culture the MSCs and to isolate the exosomes. Due to the possible loss of some vesicles during the repeated centrifugations, it is likely that the total number of isolated exosomes is lower than the actual number in the CM.

The present study provides scientific evidence of a novel osteogenic function for exosomes delivered by classically activated human monocytes to human MSCs. In the previous study, an overall effect of CM from classically activated monocytes, which possibly included both soluble factors and exosomes, resulted in a profound increase in the osteogenic gene expression in the MSCs as compared to alternatively activated or non-activated monocyte CM [21]. In a separate work, a cocktail of soluble factors, which are commonly released by classically activated monocytes, resulted in the osteogenic differentiation of MSCs [26]. In this study, we show that the osteogenic signal from the monocytes is partly delivered to the MSCs by exosomes, either packed inside and/or expressed on the delivered exosomes. Hitherto, the exact nature of the osteogenic signal, delivered via the monocyte exosomes, as well as the mode of action, is unknown. Exosomes from different cell sources, including primary cells and cell lines, are known to contain biological mediators, such as mRNA, microRNAs and proteins [27]. One possible scenario, based on the present findings, is that the ready-made molecules, mounted inside the exosome, or on its surface, are either delivered to the interior of the recipient MSCs or directly activated the MSCs by direct exosome-to-cell contact. A hypothesis of this kind can be supported by recent findings revealing that the surface of exosomes from some cell types contains Wnt molecules [28] and TGF-β [29], that are known to activate osteogenic pathways. Zhu et al. have recently shown that exosomes from MSCs enhance the expression of vascular endothelial growth factor (VEGF) by activating the extracellular signal-regulated kinase1/2 (ERK1/2) pathway [30].

Another possible mechanism involved in the revealed exosome osteogenic effect could be via the exosomal delivery of RNA. In previous studies, we have shown that exosomes from mast cells contain RNA and that the RNA profile differs between exosomes and their donor cells [16]. In the present study, it is shown that exosomes from LPS-activated monocytes contain RNA, including RNA in the size of microRNA. There is emerging evidence describing important roles for microRNAs in regulating osteogenic activity and differentiation [31], [32]. For instance, miR-335-5p (targeting Wnt inhibitor DDK1) [33], miR-196a (targeting the chondrogenic transcription factor HOXC8) [34], miR-29b (targeting inhibitors of osteoblast differentiation TGF-β3, activin receptor type-2A and beta-catenin-interacting protein 1) [35] and miR-21 (targeting the inhibitory Smad7) [36], may promote osteogenesis by down-regulating the translation of inhibitors for osteogenesis and the bone formation process. Although there is no evidence relating to the presence of these specific pro-osteogenic microRNAs in the exosomes from the classically activated monocytes, profiling studies have still shown the presence of several types of microRNA in exosomes from different cells [37], [38]. The next fundamental step will therefore be the full characterisation of the classically activated monocyte exosomes, both for the exosomal content including specific microRNAs and for surface-bound molecules expressed on the exosomes. Knowledge of this kind may provide detailed information on the molecular mechanisms whereby inflammatory and immune cells interact with mesenchymal stem cells during osteogenesis. Further, the role of exosomes in the delivery of osteoinductive cues *in vivo* for bone tissue engineering constructs and the osseointegration of implants remains to be established.

## Conclusions

The present study describes a novel pathway of signalling between human inflammatory cells (monocytes) and human mesenchymal stem cells. The results demonstrate the release of exosomes by monocytes upon stimulation by LPS, the uptake of exosomes and the promotion of osteogenic differentiation in recipient MSCs. These observations indicate a potential role for exosomes as messengers during bone regeneration. Given the possibility of selectively obtaining exosomes with osteogenic potential, further optimisation and experiments may allow for implication of such exosomes to improve the *in vivo* osteogenic response in relation to bone regeneration at biomaterials.
